# Endoscopic lateral orbitotomy

**DOI:** 10.1007/s00701-014-2205-7

**Published:** 2014-08-27

**Authors:** Tomasz Lyson, Andrzej Sieskiewicz, Marek Rogowski, Zenon Mariak

**Affiliations:** 1 Department of Neurosurgery, Medical University of Bialystok, M. Sklodowskiej-Curie 24A, 15-276 Bialystok, Poland; 2 Department of Otolaryngology, Medical University of Bialystok, M. Sklodowskiej-Curie 24A, 15-276 Bialystok, Poland

**Keywords:** Lateral orbitotomy, Endoscopy, Orbit

## Abstract

**Background:**

Lateral orbitotomy can be minimalized using contemporary endoscopy.

**Methods:**

Anatomy of the temporal fossa/orbital wall junction is described. The attachment of the temporal fascia is cut off from the orbital rim through a 1.5 cm skin incision in the lateral orbital wrinkle. The temporal muscle is detached from the bone to create a space for the telescope. An appropriate bone opening in the lateral orbital wall is created with the aid of neuronavigation to handle intraorbital pathology.

**Conclusion:**

Endoscopic lateral orbitotomy is an original alternative to the microsurgical Krönlein approach and yields good functional and cosmetic results.

**Electronic supplementary material:**

The online version of this article (doi:10.1007/s00701-014-2205-7) contains supplementary material, which is available to authorized users.

## Introduction

Lateral orbitotomy remains the most universal access to pathologies localized in the lateral orbital compartment [1, 2]. Though not extensive, it still requires a 3–4 cm skin incision, violation of the temporal muscle, resection and replacement of the orbital rim. In order to contribute to the search for a minimally invasive approach to different compartments of the orbit [3, 4], we describe here our original technique of fully endoscopic lateral orbitotomy (ELO).

### Relevant surgical anatomy

**Fig. 1:**
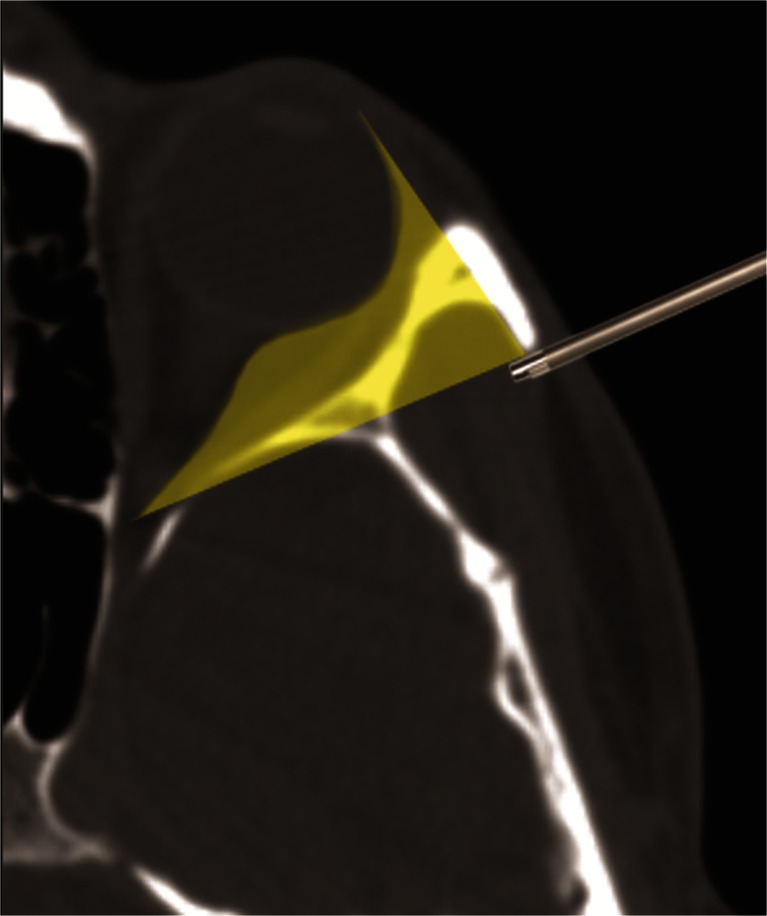
Access to the lateral orbit with endoscopic lateral orbitotomy. Shadowed area can be illuminated with an array of angled optics. With removal of particular bony structures within the shadow, a working space for straight and curved tools can be created according to need

The lateral orbital wall is formed by the zygoma, greater sphenoid wing and the frontal bone. Its outer aspect makes the deepest part of the temporal fossa. The lateral orbital rim, composed of the zygomatic process of the frontal bone and frontal process of the zygomatic bone, makes a steep anterior limit of the temporal fossa. The whole space of the temporal fossa is filled with the temporalis muscle. The most anterior fibers of the muscle, which are parallel to the orbital rim, attach directly to the bone of the temporal fossa and to the fascia temporalis. The fascia extends onto the orbital rim and turns into its periosteum. The lateral wall of the orbit escapes obliquely towards the center of the skull and its deeper half borders with the middle cranial fossa. Therefore, surgical access to the lateral orbit is possible only in its segment between the orbital rim and the dura of the middle cranial fossa. To obtain access suitable for microsurgery, one must remove the whole lateral orbital rim, as practiced in classical Krönlein orbitotomy. In contrast to microsurgery, endoscopic access can be achieved behind the orbital rim through an “artificial cavity” created by detaching the temporalis muscle from the bone and elevating it with a spatula (Fig. 1). In this way, the orbital outline and the attachment of intraorbital structures to the inner orbital rim are left intact. The extent of the orbitotomy in the coronal plane is limited by the inferior and superior orbital fissures. The inner wall of the orbit is lined by the periorbit, which is an extension of the dural sheath from the anterior cranial cavity. The periorbit is incised below or above the lateral rectus muscle, and the intraconal space can be explored up to the plane of the optic nerve.

## Methods

### Description of the technique

**Fig. 2:**
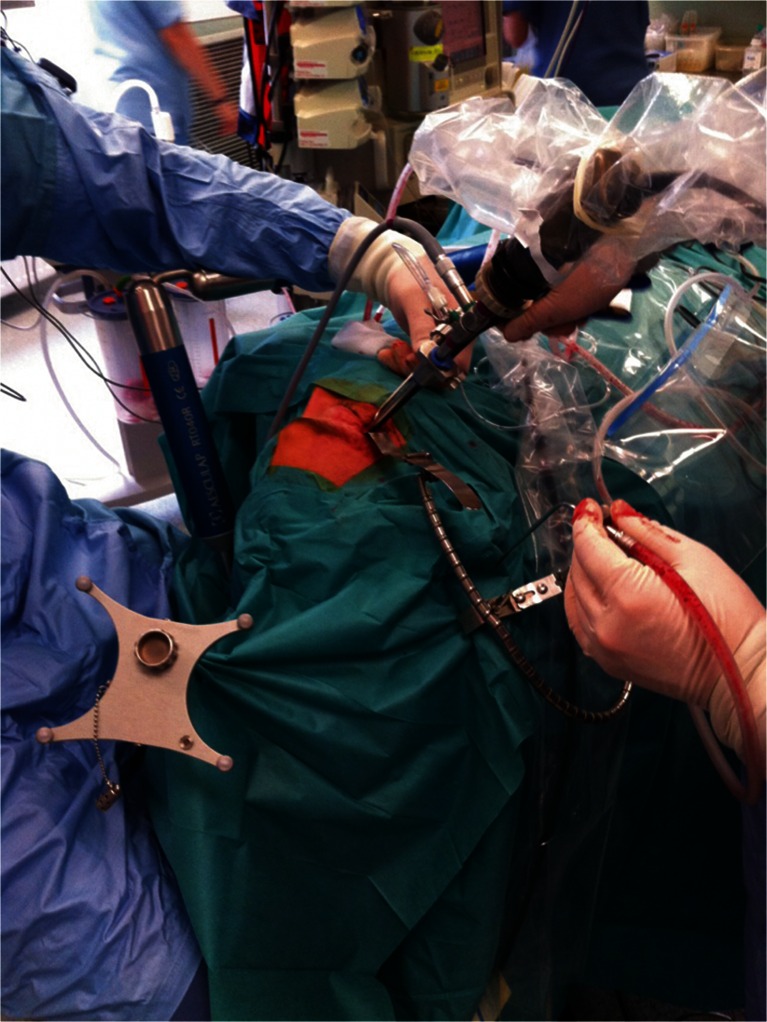
View of the surgical field with endoscope fixed to a pneumatic holder, reference frame of neuronavigation and two surgeons working with 3/4 hands technique

**Fig. 3:**
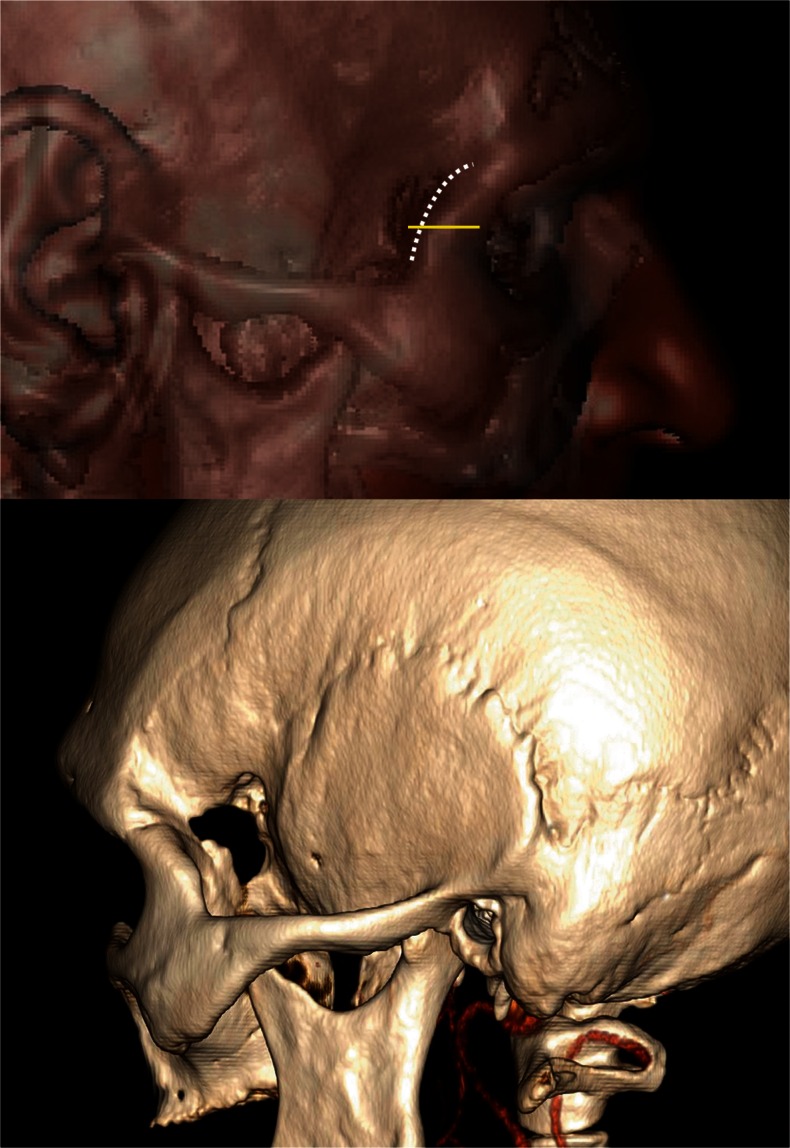
Postoperative 3D computed tomography (CT) scan showing the localization and extent of lateral orbitotomy with marked incisions: skin, horizontal line and temporal fascia, dotted line

For the procedure, the patient is positioned and draped in the same way as for a typical microscopic Krönlein orbitotomy (Fig. 2). Neuronavigation is used to localize the surgical target. Hopkins II telescopes (18 cm, ϕ 4 mm, 0°, 30°, 45°), attached to an HD camera and connected to a standard xenon light source, are used as per requirements. Manipulations are performed both with free hand technique and with the endoscope fixed to a holder. A 1.5-cm skin incision is made within a skin wrinkle, backward from a point localized about 0.5 cm behind the lateral canthus (Fig. 3). It is sufficient to place the incision only against the bony orbital rim, because the limp skin in this region can be easily mobilized backward to give access to the temporal fossa. The temporalis fascia is incised at 2.5–3 cm along the posterior edge of the orbital rim and the anterior part of the temporalis muscle is detached from the bone, thus creating a surgical corridor to the lateral orbital wall (Fig. 3). As this corridor runs between the bone and the muscle, the frontotemporal branches of the facial nerve (which run on the temporalis fascia), remain safe [5]. The space for a telescope and tools is maintained with a spatula fixed to a holder. The exact localization and the extent of the necessary bone window in the lateral orbital wall (behind the orbital rim, which is left untouched) is defined with neuronavigation (Fig. 3). Trephination itself is performed with a slightly curved pituitary high-speed drill, under visualization with a 0° telescope. From this point on, a 30° and 45° optic is used. The exposed periorbit is cut with a diamond knife. Prolapsing fat is handled with a blunt dissector and/or cauterized. The pathology is visualized, sampled for histopathological evaluation, and/or removed. The approach enables a range of manipulations within the orbit, which allows preparation along the tumor capsule, its detachment from surrounding structures, and eventually, total removal. The orbit is sealed with a patch of Tachosil®. The procedure is completed with suturing the temporal fascia to the periosteum and closure of the skin.

### Indications

**Fig. 4:**
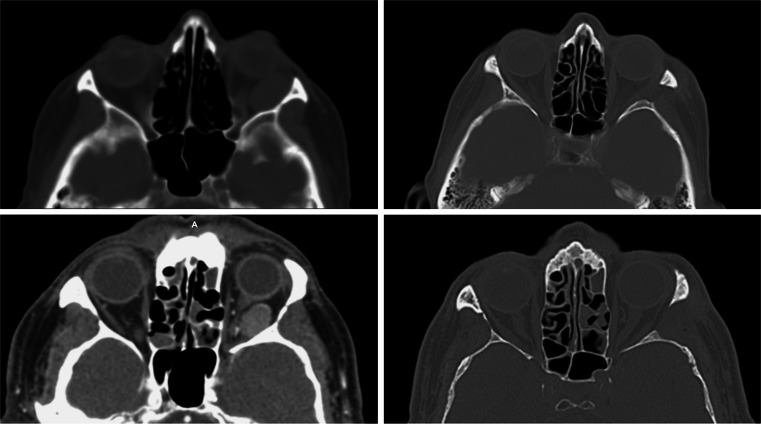
Preoperative (left) and postoperative (right) axial CT scans of two patients with intraorbital pathologies handled with endoscopic lateral orbitotomy: epithelioid hemangioma (higher) and cavernous angioma (beneath)

To date, we have used ELO for total removal of an extraconal epithelioid hemangioma (a rare, vascular tumor) and of an extra-intraconal cavernous angioma in the lateral retrobulbar space (see video and Fig. 4). ELO offers an effective surgical access to the entire lateral orbit, including the apex (Fig. 1).

### Limitations

ELO is not suitable for pathologies localized medially to the optic nerve plane nor for intraorbital masses with cranial extension. Further feasibility studies on adherent and vascular tumors are warranted.

### How to avoid complications

Neuronavigation, a full set of endoscopic tools and high proficiency in endoscopic technique are necessary prerequisites for safe and effective surgery.

### Specific peri-operative considerations

High resolution CT/magnetic resonance (MR) and registration for neuronavigation are mandatory preoperatively. Postoperatively, careful ophthalmological check-up for intraorbital hematoma/edema is obligatory.

### Specific information to give to the patient about surgery and potential risks

Patients can be informed that potential risks do not differ from the standard Krönlein procedure, including a risk of postoperative temporal muscle atrophy.

### The key points

1. With ELO, one can open the lateral orbital wall between the orbital rim and the middle cranial fossa in the sagittal plane and between the inferior and the superior corbital fissures in the coronal plane.
2. ELO enables an effective surgical access to the entire lateral orbit, including the apex.
3. ELO is not suitable for pathologies localized medially to the optic nerve plane.
4. ELO is suitable for intraorbital masses with no cranial extension.
5. A 1.5 cm skin incision is enough for opening the periosteum along the orbital rim, mobilization of the temporalis muscle and creation of a spacious endoscopic corridor to the lateral orbit.
6. The extent and localization of the bony window in the lateral orbital wall should be defined with neuronavigation.
7. The bone over the front of the middle fossa can be drilled out to obtain a more spacious approach to the orbital apex.
8. A neuronavigation system, pituitary high speed drill, an array of micro tools, holders for endoscope and spatula, as well as 0°, 30° and 45° telescopes are a minimum set of equipment.
9. Conversion to the open Krönlein approach is possible at any stage of the procedure.
10. With a limited approach, reconstruction of the orbital wall is not necessary.

## Electronic supplementary material

Below is the link to the electronic supplementary material.

ESM (MP4 21844 kb)

## References

[1] Berke RN (1953). A modified Krönlein operation. Trans Am Ophthalmol Soc.

[2] Maroon JC, Kennerdel JS (1976). Lateral microsurgical approach to intraorbital tumors. J Neurosurg.

[3] Pillai P, Lubow M, Ortega A, Ammirati M (2008). Endoscopic transconjunctival surgical approach to the optic nerve and medial intraconal space: a cadaver study. Neurosurgery.

[4] Schultheiß S, Petridis AK, El Habony R, Maurer P, Scholz M (2013). The transmaxillary endoscopic approach to the orbit. Acta Neurochir.

[5] Gosain AK, Sewall SR, Yousif NJ (1997). The temporal branch of the facial nerve: how reliably can we predict its path?. Plast Reconstr Surg.

